# Anti-VEGF drugs: evidence for effectiveness

**Published:** 2014

**Authors:** Jennifer Evans, Gianni Virgili

**Affiliations:** Lecturer: International Centre for Eye Health, London School of Hygiene and Tropical Medicine, London, UK. jennifer.evans@lshtm.ac.uk; Associate Professor: University of Florence, Florence, Italy gianni.virgili@unifi.it

**Figure:**
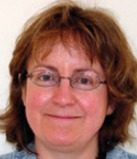
Jennifer Evans

**Figure:**
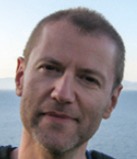
Gianni Virgili

Anti-vascular endothelial growth factors (anti-VEGF) are targeted biological drugs (e.g. monoclonal antibodies) that prevent the growth of new vessels by inhibiting VEGF. VEGF is a cytokine (cell-signalling protein) that promotes the growth of, and leakage from, new vessels. Currently there are three anti-VEGF drugs licensed for use in eye disease: pegaptanib, aflibercept, ranibizumab and one that is not licensed but is commonly used off-label (bevacizumab).

**Figure:**
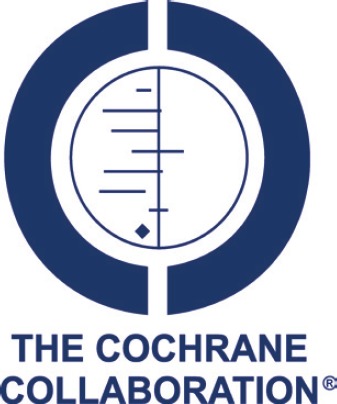


Cochrane reviews are summaries of the evidence for an effect of an intervention prepared using transparent methods that aim to reduce the risk of bias. Three features distinguish Cochrane reviews from most other systematic reviews.

1. The publication of a protocol.
2. The use of systematic and exhaustive searches of the literature.
3. Regular updating of reviews.

There are currently nine reviews and protocols in The Cochrane Library (**http://www.thecochranelibrary.com/view/0/index.html**) which addresses the effects of anti-VEGF on specific eye diseases, including one about the systemic safety of ranibizumab and bevacizumab.

This article summarises the current Cochrane reviews on proliferative (neovascular) age-related macular degeneration (AMD)^1^, diabetic macular oedema (DMO)^2^ and safety.^3^

## Participants

People enrolled in the AMD trials included in these reviews had moderate visual loss and did not have large sub-retinal haemorrhages. In real life, patients may present with more advanced disease and severe visual loss. DMO was diagnosed clinically, and OCT was usually used to confirm macular involvement. Similarly, people who enrolled in the DMO studies also had moderate visual loss.

## Effects of anti-VEGF on vision

People treated with any of the anti-VEGF agents in clinical trails more often experienced improved vision, less often lost vision, and were less likely to be legally blind than participants treated with control interventions after 1 year of treatment.

In the majority of trials of anti-VEGF for proliferative AMD, people receiving anti-VEGF were between three and ten times more likely to gain 15 or more letters of visual acuity at 1 year after treatment, and this benefit was seen at 2 years as well. They were also 80% less likely to lose 15 or more letters of visual acuity at 1 year. There were measureable benefits on quality of life.

In the DMO trials, anti-VEGF treatment was compared with laser. People receiving anti-VEGF were approximately four times more likely to gain 15 or more letters of visual acuity at 1 year compared to people who received laser treatment. They were approximately 90% less likely to lose 15 or more letters of visual acuity in one year.

Participants treated with anti-VEGF showed improvements in the structure of the back of the eye, for example central macular thickness.

## Adverse events

Inflammation and increased pressure in the eye were the most common vision-related adverse events with anti-VEGF. Endophthalmitis was reported in fewer than 1% of anti-VEGF-treated participants.

The findings on the systemic safety of ranibizumab and bevacizumab were similar; however, at the moment, most of the evidence available is for AMD, and relatively few trials have addressed this comparison for DMO.

‘Participants treated with anti-VEGF showed improvements in the structure of the back of the eye’

In the AMD and DMO reviews, the evidence was judged to be of a high quality overall.

## Systemic safety of ranibizumab and bevacizumab

One further Cochrane review has compared the systemic safety of ranibizumab and bevacizumab for neovascular AMD.^2^ In terms of systemic safety, current published and unpublished randomised controlled trials found no evidence of any difference between the two interventions.

However, the studies to date cannot definitely exclude the possibility that either treatment is more (or less) associated with harmful effects.

## Future research directions

Future research should investigate effectiveness under real-world monitoring and treatment conditions, particularly for AMD. It is possible that the same benefits may not be achieved in resource-constrained settings or in low-and middle-income countries (LMIC) where intensive diagnostic follow-up is difficult and under-treatment may be more likely than in industrialised countries. Differences between drugs are also of interest, especially comparing low cost off-label bevacizumab to licensed drugs in diabetic retinopathy and DMO. This is an important public health issue in LMIC, since efficacy has been demonstrated to be similar for AMD. For all these drugs and indications, safety in high-risk populations, particularly regarding cardiovascular risk, proved to be good, but has to be investigated further.

**Figure:**
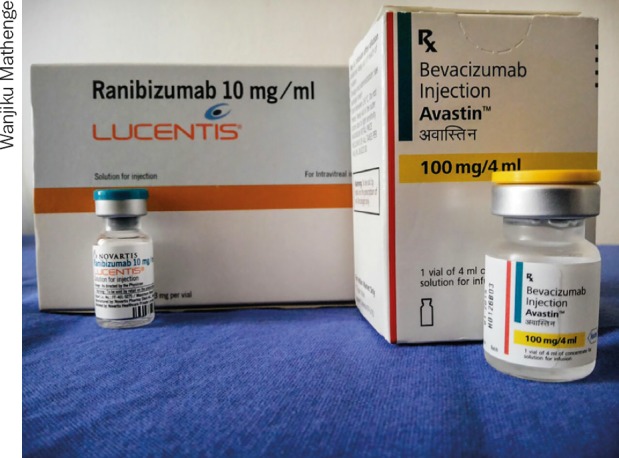
Commonly used anti-VEGF drugs
